# Inhibiting Effect of Electroacupuncture at Zusanli on Early Inflammatory Factor Levels Formed by Postoperative Abdominal Adhesions

**DOI:** 10.1155/2014/950326

**Published:** 2014-08-12

**Authors:** Lijian Zhang, Huizhen Wang, Zhenjun Huang, Xian Shi, Sen Hu, Ingrid Gaischek, Daniela Litscher, Lu Wang, Gerhard Litscher

**Affiliations:** ^1^Department of Rehabilitation of the 309th Hospital of People's Liberation Army, Beijing 100091, China; ^2^Department of Acupuncture and Moxibustion, Military Acupuncture Training Centre, People's Liberation Army General Hospital, No. 28, Fuxing Road, Beijing 100853, China; ^3^Laboratory of Shock and Organ Dysfunction, Burns Institute, First Affiliated Hospital of PLA General Hospital, Beijing 100037, China; ^4^Research Unit for Complementary and Integrative Laser Medicine, Research Unit of Biomedical Engineering in Anesthesia and Intensive Care Medicine, TCM Research Center Graz, Medical University of Graz, Auenbruggerplatz 29, 8036 Graz, Austria

## Abstract

We observed the inhibitive effect of electroacupuncture (EA) at Zusanli on inflammatory mediators of postoperative intra-abdominal adhesions to find out the relationship between EA and the cholinergic anti-inflammatory pathway. Sixty-four rats were divided into 8 groups (A–H, each = 8): A = sham control; B = abdominal adhesions model; C = abdominal adhesions plus EA; D = sham acupoint control; E = abdominal adhesions plus vagotomy; F = abdominal adhesions plus EA after vagotomy; G = abdominal adhesions plus *α*-bungarotoxin (BGT); and H = abdominal adhesions plus EA after *α*-BGT. *α*-BGT (1 *μ*g/kg) was injected into the abdominal cavity after surgery, and the bilateral celiac vagotomy was done during the surgery. On the third day the levels of inflammatory mediators (TNF-*α*, nitric oxide (NO), and nitric oxide synthase (NOS)) in tissues were evaluated. The abdominal adhesion groups developed obvious edema. Compared with sham control, the abdominal adhesion resulted in a significant elevation of inflammatory mediators. EA lowered the elevated levels of inflammatory mediators significantly; EA plus *α*-BGT and vagotomy showed less anti-inflammatory effects. The activation of the cholinergic anti-inflammatory pathway might be one of the mechanisms of EA at Zusanli acupoints to exert the anti-inflammatory effects.

## 1. Introduction

Abdominal adhesion is a common complication in pelvic surgery, with an incidence of up to 90%. Severe cases can lead to intestinal obstruction and female infertility, thus bringing great suffering and a heavy burden to the patient [1]. For ischemia, injury, and foreign body irritation during surgery, large amounts of proinflammatory cytokines in tissues are produced, which can lead to an increase of vascular permeability and angiogenesis in the adhesion sites, resulting in abdominal adhesions [2]. Therefore, an early lowering of abnormally elevated proinflammatory cytokine levels is one of the main ways to prevent and treat abdominal adhesions.

Acupuncture treatment has a wide range of clinical applications. References show that electroacupuncture at the Zusanli (ST36) acupoint has significant inhibiting effects on sepsis and endotoxemia with proinflammatory cytokine levels, which can effectively protect organ function [3, 4].

The aim of this study was to observe the effects of electroacupuncture (EA) at the Zusanli acupoint on early inflammatory cytokines formed by postoperative abdominal adhesion and to explore the anti-inflammatory mechanism of Zusanli.

## 2. Materials and Methods

### 2.1. Experimental Animals and Grouping

Sixty-four male Wistar rats, weighing 220 ± 20 g, were obtained from the Animal Center of Military Medical Sciences (Certificate number SXCL-(Army-2007-004)). The rats were housed at a constant temperature of 24 ± 2°C and constant humidity (50 ± 5%) conditions for one week. They received no food in the 12 hours before the experiment but had free access to water.

The rats were randomly divided into 8 groups (each = 8): group A (sham control), group B (abdominal adhesions model), group C (abdominal adhesions plus EA), group D (sham acupoint control), group E (abdominal adhesions plus vagotomy), group F (abdominal adhesions plus EA after vagotomy), group G (abdominal adhesions plus *α*-bungarotoxin (BGT)), and group H (abdominal adhesions plus EA after *α*-BGT).

### 2.2. Experimental Methods

*(1) Model Making*. The method described by Chiang et al. [5] was used to achieve the rats abdominal adhesions model. After successful intramuscular anesthesia using ketamine plus Sumianxin (2 : 1 preparation, 0.5 mL/kg), the rats were fixed in supine position. The skin was prepared routinely and disinfected, and sterile towels were prepared. After cutting 2 cm along the abdominal midline, the cecum was carefully pulled out and fricted to bleed by sterile dry gauze, with an area of approximately 2 cm × 1 cm. Number 0 silk was used to fix a 1 cm × 1 cm medical silicone membrane, trying not to damage other tissues. The cecum was inserted back into the abdominal cavity, and the abdomen was closed with number 0 silk. Rats had free access to water and food after surgery.

*(2) Processing Methods*. For group A, the cecum was flipped and the abdomen closed without any special treatment. In groups B–F, abdominal adhesions surgery was conducted. For groups C, F, and H, about 40 minutes was spent before the rats were awake after abdominal adhesions surgery. Then they were fixed with a bag (limbs exposed through four holes), and the bilateral Zusanli acupoint (outside of the knee, about 5 mm below the fibular head) [6] was selected and acupunctured. The two needles were connected with two electrode coils, and the acupoint was stimulated continuously (2 mA, 2–100 Hz) for 1 hour with an EA device (domestic HANS, LH202H). For group D, a nonacupoint (about 5 mm below the outer of Zusanli acupoint) was stimulated using the same electrode, parameters of intensity, frequency, and time (2 mA, 2–100 Hz, 1 h). For groups E and F, the bilateral abdominal vagus nerve was precut before modeling. The *α* 7 receptor-specific antagonist *α*-BGT (1 *μ*g/kg) was immediately injected into abdominal cavity after surgery. After surgery, the rats had free access to water and food and were sacrificed on the third day.

### 2.3. Observations and Detection Methods

On the third day, all rats were fixed after intramuscular anesthesia by ketamine plus Sumianxin (2 : 1 preparation, 0.5 mL/kg), and the abdominal cavity of those was cut again. The rats were sacrificed by draining abdominal aortic blood. A part of appendix was clipped and was packed in liquid nitrogen cryopreservation. 100 mg appendix and 9 mL saline were added to prepare a 10% homogenate. Then it was centrifuged for 10–30 min with a speed of 1000 and 3000 rev/min, respectively (the speed depends on the measurement targets). The supernatant after centrifuging was cryopreserved (−80°C) and prepared to be detected.

*(1) TNF-*α* Levels*. A rat TNF-*α* ELISA test kit (Diaclone Company) was used (sensitivity 20 pg/mL, detection range 20–1000 pg/mL). In strict accordance with the manual steps, the instrument automatically calculated a standard curve regression equation: *y* = 0.0173 + 0.0003*x*, *r* = 0.9942. The optical density of the sample was put into a standard curve and multiplied by the appropriate dilution factor to calculate the sample's TNF-*α* content, with a unit of pg/mL. 

*(2) Detection of Nitric Oxide (NO) Content*. An NO kit (Nanjing Jiancheng Bioengineering Institute) was used in strict accordance with the instructions; the results are given in *μ*mol/gprot unit.

The formula is NO content (*μ*mol/gprot) = (absorbance measurement tube − tube blank absorbance)/(standard Quasi absorbance tube − absorbance of blank tube) × standard concentration (20 *μ*mol/L) × sample protein content (gprot/L). 

*(3) Detection of Nitric Oxide Synthase (NOS) Content*. We used an NOS kit (Nanjing Jiancheng Bioengineering Institute) in strict accordance with the instructions; results are given in U/gprot unit.

The formula is Total NOS activity = (total NOS tube optical density value measured − blank tube optical density)/coloring matter nanomolar extinction coefficient × reaction liquid total volume/sample volume × 1/(optical path × reaction time) ÷ 1000.

### 2.4. Statistical Analysis

ANOVA statistical analysis software was used to process the data. Differences between each group were compared, indicated with $\left(\overset{-}{x}\pm s\right)$. A *P* value of <0.05 was considered statistically significant.

## 3. Results

### 3.1. TNF-*α* Levels

On the third postoperative day, group A showed a very low content of tissue TNF-*α*, significantly lower than the other groups (*P* < 0.01). Compared with group C, groups B, D, E, F, G, and H had a significantly increased TNF-*α* content (*P* < 0.01 or *P* < 0.05), which shows that EA was able to significantly reduce early tissue TNF-*α* levels of abdominal adhesion but not to reach to the level of Group A. The TNF-*α* levels of groups B, D, E, F, G, and H showed no significant difference (*P* > 0.05) between them (see Table 1).

### 3.2. NO Content

On the third postoperative day, all groups showed a significantly increased NO content (*P* < 0.01) compared with group A. Group C had a significantly lower NO content than groups B, D, E, F, G, and H (*P* < 0.01 or *P* < 0.05). Groups B and D, groups E and F, and groups G and H had no significant differences (*P* > 0.05) (see Table 2).

### 3.3. NOS Content

The rats that had undergone intraperitoneal adhesions surgery had a higher NOS content (*P* < 0.01). Group C showed a lower NOS content than groups B, D, E, F, G, and H (*P* < 0.05). Compared with group B, groups D, E, F, G, and H had no significant NOS content difference (*P* > 0.05) (see Table 3).

## 4. Discussion

Abdominal and/or pelvic surgery can lead to peritoneal injury and tissue inflammation, which can produce large amounts of proinflammatory cytokines. These proinflammatory cytokines can increase vascular permeability to deposit fibrin and promote angiogenesis and remodeling, thus triggering the formation of abdominal adhesions [7]. Tumor necrosis factor-*α* (TNF-*α*), nitric oxide (NO), nitric oxide synthase (NOS), and other cytokines are involved in inflammation and play an important role in the formation of abdominal adhesions [8]. Thus, early suppression of abnormally elevated proinflammatory cytokine levels is one of the effective means for preventing and treating postoperative abdominal adhesions.

TNF-*α* is the cytokine-mediated inflammation. In cases of endotoxemia, early sepsis, and burns, plasma or tissue TNF-*α* levels were found to be significantly increased, adding to the pathological organ tissue damage [9–12]. Saba et al. found that TNF-*α* levels were significantly associated with the adhesion degree after abdomen surgery, which can be used as a reliable indicator of human biology postoperative peritoneal adhesion formation [13].

In the formation process of tissue adhesions, proinflammatory cytokines can cause a variety of tissues and cells of inducible nitric oxide synthase (iNOS) expression, catalyzing a large amount of catalytic NO, causing a series of pathological responses. iNOS plays an important role in systemic infection and excessive pathophysiology of inflammatory response. Hu et al. [3, 10–12] conducted a series of experiments and found that in rats with severe burns, endotoxemia, and early-stage sepsis, plasma and tissue NOS activity increased, causing a higher NO production leading to multiple organ disorders.

According to traditional Chinese medicine, the pathological bases of postoperative abdominal adhesions are meridian injuries, blood stasis, damp stagnation, and gastrointestinal inhibition, resulting in healthy-qi deficiency and pathogens sufficiency.

Acupuncture points of Yang Ming meridians and abdomen are mainly used in clinical care. Zusanli is the confluent acupoint of Yang Ming meridians, which can promote gastrointestinal motility, improve the blood flow and immune system and can have other effects; in addition, modern research results show that Zusanli has anti-inflammatory effects, which can reduce inflammatory factor levels abnormally elevated in blood plasma or tissue of the sepsis, endotoxin, and scald rats, inhibiting organ dysfunction [9–12].

In our experiment we found that in the early postoperative abdominal adhesion formation, cecums got to be edema. TNF-*α*, NO, and NOS were significantly increased and thus priming the immune and inflammatory cascade. Group C showed lower levels of inflammatory cytokines, indicating that acupuncture at the Zusanli acupoint can reduce abnormally elevated levels of inflammatory factors, reducing the pathological organ tissue damage. In a series of experiments on peritoneal adhesions, we observed that EA at Zusanli can significantly inhibit the formation of adhesions in abdominal tissue angiogenesis, reducing inflammatory infiltration, and thus effectively prevent abdominal adhesion formation [14, 15].

The cholinergic anti-inflammatory pathway goes by the central nervous system and the cholinergic neurotransmitter (acetylcholine, Ach). The Ach can significantly inhibit the release of endotoxin-stimulated human blood TNF-*α* and IL-6 and other proinflammatory cytokines. In addition, direct electrical stimulation of cholinergic nerves (efferent vagus nerve) can inhibit the synthesis of TNF [16]. The cholinergic nerves mainly undergo functions through both M and N receptors (M and N in the presence of multiple receptor subtypes). The excitatory cholinergic anti-inflammatory effects of the molecular basis of choline are present in the N-type surface of the macrophage receptor *α*_7_ subunit (*α*7nAChR) [17]. Torres-Rosas et al. found that sciatic nerve activation with electroacupuncture controlled systemic inflammation and rescued mice from polymicrobial peritonitis, which induces vagal activation of aromatic L-amino acid decarboxylase, leading to the production of dopamine in the adrenal medulla [18]. For rats with endotoxemia and sepsis, a precut bilateral abdominal vagus nerve or injection of *α*7nAChR blocks *α*-BGT; the anti-inflammatory and organ protective effects of EA at Zusanli diminished or disappeared, which resulted in the inflammatory factor levels of plasma or tissue inflammatory elevated [4, 9, 11]. In this experiment, we found the cecum treated was getting inflammation and edema. A part of cecum was clipping in order to observe and detect the inflammatory response. By researching the levels of inflammatory cytokines in tissues, we plan to detect inflammatory factors in plasma and study the systemic inflammatory response.

In our experiment, we found that in the early phase of postoperative abdominal adhesion formation, the proinflammatory cytokine levels produced by precut bilateral abdominal vagus nerves or tissue treated with *α*-BGT showed no significant differences (*P* > 0.05) compared with the model group. The implementation of EA at Zusanli had no significant anti-inflammatory effects.

Zusanli acupuncture can significantly reduce inflammatory cytokines levels of postoperative abdominal adhesions, inhibit the activated immune and inflammatory cascade, and prevent abdominal adhesion formation. Precutting abdominal vagus or giving N receptor blocking agent *α*-BGT treatment can lead to the disappearance of the anti-inflammatory effects of EA at Zusanli. We believe that EA at Zusanli can effectively inhibit the interaction of early postoperative abdominal adhesion formation mechanism and excite the cholinergic anti-inflammatory pathway.

## Figures and Tables

**Table 1:** Comparison of cecum TNF-*α* levels [($\overset{-}{x}\pm s$), Pg/gprot].

Groups	Number of animals	TNF-*α*	*P* value compared with group A	*P* value compared with group C
Group A	8	79.73 ± 20.44	—	0.0000
Group B	8	648.30 ± 114.06	0.0000	0.0366
Group C	8	509.29 ± 126.27	0.0000	—
Group D	8	662.12 ± 100.88	0.0000	0.0181
Group E	8	713.92 ± 114.83	0.0000	0.0044
Group F	8	652.43 ± 136.54	0.0000	0.0471
Group G	8	698.15 ± 122.76	0.0000	0.0089
Group H	8	664.26 ± 125.52	0.0000	0.0274

**Table 2:** Comparison of cecum NO levels [($\overset{-}{x}\pm s$), Mmol/gprot].

Groups	Number of animals	NO	*P* value compared with group A	*P* value compared with group C
Group A	8	1.06 ± 0.28	—	0.0000
Group B	8	2.69 ± 0.44	0.0000	0.0286
Group C	8	2.28 ± 0.18	0.0000	—
Group D	8	2.67 ± 0.37	0.0000	0.0179
Group E	8	2.81 ± 0.36	0.0000	0.0023
Group F	8	2.74 ± 0.29	0.0000	0.0019
Group G	8	2.66 ± 0.25	0.0000	0.0036
Group H	8	2.62 ± 0.17	0.0000	0.0017

**Table 3:** Comparison of cecum NOS activity [($\overset{-}{x}\pm s$), U/gprot].

Groups	Number of animals	NOS	*P* value compared with group A	*P* value compared with group C
Group A	8	1.11 ± 0.26	—	0.0000
Group B	8	2.35 ± 0.21	0.0000	0.0215
Group C	8	2.00 ± 0.32	0.0000	—
Group D	8	2.46 ± 0.42	0.0000	0.0273
Group E	8	2.52 ± 0.41	0.0000	0.0134
Group F	8	2.40 ± 0.18	0.0000	0.0081
Group G	8	2.42 ± 0.28	0.0000	0.0144
Group H	8	2.38 ± 0.26	0.0000	0.0207
